# An audit of the establishment of a cardiac magnetic resonance imaging service in a public tertiary hospital setting in the Western Cape Province of South Africa

**DOI:** 10.11604/pamj.2024.49.15.44617

**Published:** 2024-09-12

**Authors:** Carien van Schalkwyk, Beulah Christina van Zyl, Phillipus George Herbst, Christelle Ackermann

**Affiliations:** 1 Department of Radiology, Tygerberg Hospital, Stellenbosch University, Stellenbosch, South Africa,; 2 Department of Cardiology, Tygerberg Hospital, Stellenbosch University, Stellenbosch, South Africa

**Keywords:** Heart, magnetic resonance imaging, cardiovascular diseases, clinical audit, Africa South of the Sahara

## Abstract

**Introduction:**

cardiovascular magnetic resonance imaging is considered the standard of care for many clinical cardiovascular applications. Magnetic resonance imaging is a scarce resource in sub-Saharan Africa, with a paucity of cardiac magnetic resonance imaging clinical services and research. The aim of this audit was to review the cardiac magnetic resonance imaging service provided at a public tertiary hospital in Cape Town, Western Cape Province, South Africa.

**Methods:**

a retrospective, descriptive audit via quantitative record review of Tygerberg Hospital´s cardiovascular magnetic resonance imaging service was conducted from the inception thereof on 1^st^ April 2015 up to 31^st^ October 2022.

**Results:**

a total of 1,403 cardiovascular magnetic resonance imaging scans met the inclusion criteria. The mean age of the study population was 43 years, and 52% were female. The most common patient comorbidities were modifiable cardiovascular risk factors, including hypertension (22%; n=306), cigarette smoking (9.6%; n=134), diabetes mellitus type II (6.7%; n=94) and dyslipidaemia (4.4%; n=62). Sixty-three percent (n=888) of scans were performed after hours. In 93% of scans, intravenous gadolinium-based contrast agents were administered. Nonischaemic cardiomyopathy dominated the indications (56.7%; n=976) and final diagnosis (42%; n=589). The most common incidental extracardiac finding was hilar or mediastinal lymphadenopathy (6%; n=82).

**Conclusion:**

the recently established, functional cardiovascular magnetic resonance imaging service at Tygerberg Hospital serves a unique patient population with a comparatively differently distributed cardiac disease spectrum, contributing to research diversity.

## Introduction

Cardiovascular disease is the leading contributor to global mortality, with 80% of those deaths occurring in low- and middle-income countries. The earlier age of onset of cardiovascular disease in sub-Saharan Africa is of great concern. The epidemiological transition is rapidly resulting in an increase in noncommunicable diseases, straining already overburdened healthcare services [1-3].

Clinical magnetic resonance imaging (MRI) was introduced in the 1980s. Technological advances over the past 40 years have more recently allowed for the acquisition of extraordinary-quality images of the beating human heart. This has revolutionized modern practice in cardiology [4]. Cardiovascular magnetic resonance imaging (CMR), sometimes known as cardiac MRI, accurately and noninvasively assesses cardiac function, tissue characteristics, perfusion, and valves. Cardiovascular magnetic resonance imaging has an extensive range of clinical applications, improves diagnostic accuracy, and allows risk stratification, all enhancing patient outcomes [5]. However, MRI is a scarce resource on the African continent with the smallest number of MRI services per population, globally [6]. More than two-thirds of African countries have less than one MRI machine per million of the population [6]. Multicentre CMR registries such as the European Cardiovascular Magnetic Resonance (EuroCMR) registry and the Global Cardiovascular Magnetic Resonance Registry (GCMR) have been established to standardize CMR services [7,8]. However, in Africa, there is a paucity of published data on CMR [6]. Hence, establishing CMR clinical services and research in sub-Saharan Africa needs to be addressed with careful consideration of the paucity of resources.

Given the great value of CMR, a CMR service was introduced at Tygerberg Hospital in Cape Town, Western Cape Province, South Africa in April 2015 to support clinical diagnosis and to initiate CMR-directed research. Collaboration between the cardiology and radiology departments was the key to success in establishing a functional CMR service. The constraint of a single 1.5 Tesla MRI scanner serving a large population at Tygerberg Hospital necessitated increased scanning capacity by scanning elective cases after-hours. The aim of this audit was to determine patient population characteristics, CMR procedure, indications, final diagnosis, diagnostic impact, and incidental extracardiac findings with regard to the CMR service at Tygerberg Hospital since its inception on 1^st^ April 2015 up to 31^st^ October 2022.

## Methods

**Study design and setting:** a retrospective, descriptive audit via quantitative record review of CMR scans was conducted at Tygerberg Hospital, a tertiary teaching hospital in Cape Town, South Africa to determine patient population characteristics, CMR procedure, indications, final diagnosis, diagnostic impact and incidental extracardiac findings with regard to the CMR service. Tygerberg Hospital is the second largest hospital in South Africa and receives referrals from the greater eastward metropolitan expansion and surrounding rural districts, ultimately serving a population of 3,784,533 [9].

**Study population:** the target population included all patients undergoing CMR at Tygerberg Hospital during the study period. Consecutive sampling was done of all clinical CMR scans performed between 1^st^ April 2015 and 31^st^ October 2022. Clinical CMR scans that were diagnostically inadequate or whose written report was lacking were excluded from the study.

**Data collection:** the data source was CMR reports on the Radiology Picture Archiving Communication System (PACS) and Tygerberg Hospital electronic local health records. A structured electronic data collection tool was developed by the principal investigator. All variables assessed, including definitions and classifications, were predefined. Potential confounders were also considered. Demographic variables included age and gender. The principal investigator read the report on PACS and collected and classified data on the time when the scan was performed, whether a gadolinium-based contrast agent (GBCA) was administrated, and the reporting structure, namely the primary reader, the radiologist, the cardiologist, or both. The clinical indication and patient comorbidities were classified based on the history provided by the referring clinician on the request. The final diagnosis and incidental extracardiac findings were extracted and classified. Electronic local health records were used to correlate the diagnosis from the CMR report with the final diagnosis, based on clinical findings and other investigations.

An assessment was also made of the diagnostic impact of each CMR scan. The indication (i.e. initial diagnosis) was compared to the final diagnosis and classified as follows: 1) a new diagnosis that was not suspected was made; 2) a suspected diagnosis was confirmed; 3) the diagnosis did not change, but valuable findings influenced management; 4) the scan was noncontributory.

The data collection tool was pretested and refined with a sample of 10% of the total sample. Epidata entryClient software version 4.6.0.6. was used to formulate the data collection tool. Data were extracted into a Microsoft Excel spreadsheet in which data cleansing and analysis were performed. The spreadsheet was stored on a password-protected computer, and only the investigators had access to the data.

**Definitions:** clinical indication and final diagnosis categorical classification were based on the American College of Radiology Appropriateness criteria and the American Heart Association classification of cardiomyopathies, respectively.

**Statistical analysis:** only descriptive statistics was done, and no formal hypothesis testing was performed. Numerical data were described with mean, median, range, and standard deviation. Categorical variables were described using frequencies and percentages. Data analyses were performed with Microsoft Excel software under the supervision of the Department of Biostatistics at Stellenbosch University. The collected data are available from the corresponding author on request.

**Ethical considerations:** ethical approval for the study was granted by the local ethics committee, the Health Research Ethics Committee (HREC) of Stellenbosch University (S23/12/319), and site approval was obtained from the hospital. As this was a retrospective descriptive study, a waiver of consent was granted by the HREC. Each patient was allocated an anonymous code, and confidentiality was maintained. All data were stored on a password-protected computer.

## Results

**General characteristics of the study population:** a total of 1,403 CMR scans were included for the study period, while 337 CMR scans were excluded due to no written report available (n=322), nondiagnostic imaging secondary patient factors (n=11) or technical factors (n=4). Of the 322 CMR scans with no reports, 265 were performed for research purposes. Clinically relevant CMR scans also performed for research purposes were included in the sample. The mean age of patients in this audit was 43 years (standard deviation 15.8 years; range 3-87 years), and 52% (n=724) were female. The most common patient comorbidities were modifiable cardiovascular risk factors, including hypertension (22%; n=306), cigarette smoking (9.6%; n=134), diabetes mellitus type II (6.7%; n=94), dyslipidaemia (4.4%; n=62) and obesity (0.4%; n=6). Significant communicable diseases included the human immunodeficiency virus (4.8%; n=68) and tuberculosis (1.8%; n=20).

**Cardiovascular magnetic resonance imaging (CMR) procedure:** sixty-three percent (n=888) of CMR scans were performed after hours (Monday to Friday, 4 pm to 8 am, and weekends). Intravenous GBCA was administered in most of the cases (93%; n=1300). The CMR scans were reported by both a radiologist and cardiologist (43%; n=606), a cardiologist (2%; n=133) or a radiologist (54%; n=764).

**Figure 1:**
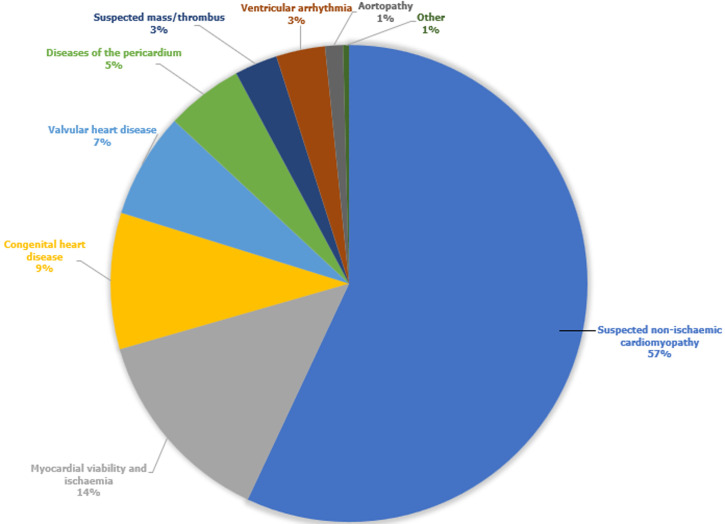
primary indications of reported clinical cardiac magnetic resonance imaging scans performed at Tygerberg Hospital, a public tertiary institution in Cape Town, South Africa from 1^st^ April 2015 to 31^st^ October 2022 (n=1403)

**Indication for cardiovascular magnetic resonance imaging and final diagnosis:** the most frequent indication for CMR was suspected nonischaemic cardiomyopathy (56.7%; n=976), including myocarditis (23.6%; n=331), followed by myocardial ischaemia and viability (13.5%; n=189) (Figure 1). The frequency of the final diagnosis of nonischaemic cardiomyopathy (42%; n=589) overshadowed that of ischaemic cardiomyopathy (17%; n=239). The main contributors to nonischaemic cardiomyopathy were myocarditis (24.6%; n=145), hypertensive heart disease (10.2%; n=60), and hypertrophic cardiomyopathy (10.4%; n=61). More than half (53%; n=47) of the diseases of the pericardium were attributable to constrictive pericarditis (Table 1).

**Table 1:** final diagnosis categorical classification of reported clinical cardiac magnetic resonance imaging scans performed at Tygerberg Hospital, a public tertiary institution in Cape Town, South Africa from 1^st^ April 2015 to 31^st^ October 2022 (n=1403)

Final diagnosis categories	n	Percentage (%)
Nonischaemic cardiomyopathy	589	42
Ischaemic heart disease	239	17
Normal	149	10.6
Congenital heart disease	131	9.3
Valvular disease	101	7.2
Diseases of the pericardium	89	6.3
Cardiac masses	15	1.1
Vascular diseases	20	1.4
Miscellaneous	70	5

**Figure 2:**
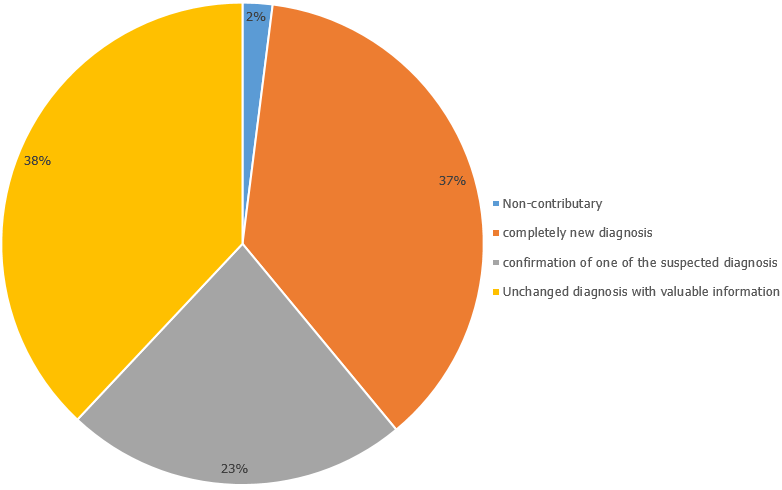
diagnostic impact of reported clinical cardiac magnetic resonance imaging scans performed at Tygerberg Hospital, a public tertiary institution in Cape Town, South Africa from 1^st^ April 2015 to 31^st^ October 2022 (n=1403)

**Diagnostic impact and incidental extracardiac findings:** cardiovascular magnetic resonance imaging had a positive diagnostic impact in the majority of cases, whereas a new diagnosis that was not suspected was made in 37% (n=520) of cases, a suspected diagnosis was confirmed in 23% (n=327) of cases and the diagnosis did not change but valuable findings influenced management in 38% (n=532) of cases (Figure 2). Thirty-eight percent (n=520) of patients had extracardiac findings related to cardiac failure, including pleural effusions (22%; n=303), atelectasis (14%; n=192), and passive hepatic congestion (2%; n=23). The most common significant incidental extracardiac finding was hilar or mediastinal lymphadenopathy (6%; n=82) (Table 2).

**Table 2:** incidental extracardiac findings of reported clinical cardiac magnetic resonance imaging scans performed at Tygerberg Hospital, a public tertiary institution in Cape Town, South Africa from 1^st^ April 2015 to 31^st^ October 2022 (n=1403)

Incidental extracardiac finding	n	Percentage (%)
Pleural effusion	303	22
Atelectasis	192	14
Passive hepatic congestion	25	2
Lymphadenopathy	82	6
Pulmonary nodules or masses	30	2
Pulmonary fibrosis	23	2
Liver lesion	34 (29 benign)	2
Renal pathology	29 (24 benign)	2
Osseous abnormalities	10 (8 benign)	1
Biliary cholelithiasis	5	0.4
Splenic lesions (all benign)	11	0.8
Hiatus hernia	9	0.6
Adrenal lesion (non-benign)	4	0.3
Breast lesion (benign)	1	0.1

## Discussion

The aim of this audit was to determine patient population characteristics, CMR procedure, indications, final diagnosis, diagnostic impact, and incidental extracardiac findings with regard to the CMR service at Tygerberg Hospital.

The mean age of patients in this audit is 43 years (SD 15.8 years), two decades lower in comparison to high-income countries [2]. This is relevant, indicating that cardiac disease affects the financially productive age group in our sample and highlights the value of the CMR service [10]. The near-equal gender distribution in this audit is probably due to a selection bias.

The prevalence of hypertension in our study (22%; n=306) is comparable to the estimated 30% of Africans who suffer from hypertension; however, this finding may be due to secondary selection bias [11]. All referrals for CMR were from the Department of Cardiology, after primary assessment and work-up, which may have included echocardiograms and invasive coronary artery catheterization, resulting in preselecting indications and expected pathology. The significance of communicable diseases in our setting is emphasized by the World Health Organization´s 2023 global tuberculosis report according to which Africa has the highest estimated number of incident tuberculosis cases globally in addition to human immunodeficiency virus coinfection exceeding 50% in southern Africa [12].

Sixty-three percent (n=888) of scans were performed after hours, pointing to the effective utilization of the single MRI scanner at Tygerberg Hospital in terms of maximizing imaging capacity, even when challenged with competing emergency MRI scans. Most CMR scans used intravenous GBCA (93%), similar to the EuroCMR registry and the GCMR [7,8], pointing to its utility in distinguishing among various cardiomyopathies, assessing myocardial viability, and doing risk stratification [13]. Up to date, no documented contrast-related complications with CMR scans have occurred at our institution. Forty-three percent (n=606) of scans were formally reported by both a cardiologist and a radiologist. However, all CMR scans were conducted under the direct supervision of a cardiology research fellow or consultant and reported in consultation with the Department of Cardiology. This all emphasizes the value of a collaborative approach to CMR and is also reflected by the EuroCMR audit [7].

The indication of the majority of CMR scans was for suspected nonischaemic cardiomyopathy. This is in stark contrast with the EuroCMR registry and the GCMR, according to which most scans were performed for myocardial ischaemia and viability, indicating a unique population of cardiac patients in our setting [7,8]. The final diagnosis was dominated by nonischaemic cardiomyopathy (42%; n=589). This reflects the utility of CMR in the diagnosis of myocarditis, which until very recently still depended on invasive myocardial biopsy. In contrast, the workup of ischaemic heart disease and its complications has well-established protocols with CMR only indicated in selected cases. Historically, the most common cause of heart failure in sub-Saharan Africa has been nonischaemic cardiomyopathies, in contrast to ischaemic heart disease in high-income countries [14-16]. However, the prevalence of ischaemic heart disease in sub-Saharan Africa is rapidly increasing [10].

**Figure 3:**
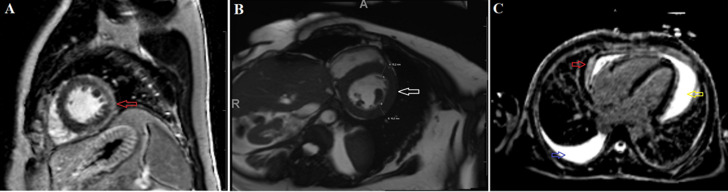
cardiac MRI examples of cardiac diseases that occurred in the audit: A) myocarditis: subepicardial delayed gadolinium enhancement (red arrow) of the anterolateral, posterior, and inferior left ventricular wall on a high-resolution short-axis phase-sensitive inversion recovery sequence is consistent with myocarditis according to the Lake Louis 2018 criteria; B) hypertensive heart disease, concentric left ventricular wall hypertrophy (white arrow) on a short-axis steady-state free precession sequence; C) tuberculous pericarditis confirmed on pericardial fluid aspirate, a large pericardial effusion (yellow arrow) with a thickened and enhancing pericardium (red arrow) on a four-chamber delayed gadolinium enhancement phase-sensitive inversion recovery sequence is consistent with acute tuberculous pericarditis; a right pleural effusion is also present (blue arrow)

The most common disease entity contributing to nonischaemic cardiomyopathy was myocarditis (Figure 3A), followed by hypertensive heart disease (Figure 3B) and hypertrophic cardiomyopathy. In concordance with high-income countries, the most common aetiology of myocarditis at our institution is a viral infection (Parvovirus B19) [17]. Longstanding poorly controlled hypertension leads to cardiac injury and ultimately hypertensive heart disease, explaining the significance thereof [11]. The clinical utility of CMR in hypertrophic cardiomyopathy is multifactorial by means of distinguishing hypertrophic cardiomyopathy from other common phenocopies, doing risk stratification, and providing follow-up [18].

The frequency of congenital heart disease was higher in our audit (9%; n=131) in comparison with the EuroCMR registry (2.2%) and the GCMR (4%). This may be a selection bias due to referrals from the Grown-Up Congenital Heart Disease (GUCH) clinic at Tygerberg Hospital but does point to the high number of adults with congenital heart disease in our setting. Cardiovascular magnetic resonance imaging uses non-ionising radiation and is a useful imaging adjunct to echocardiography, aiding in surgical planning and postoperative follow-up for complex cardiac surgical repairs; hence, its role is pivotal at our institution [19].

In comparison with the EuroCMR registry and the GCMR, a larger number of pericardial diseases (6%; n=89) were found in this audit. The most frequent pericardial disease was constrictive pericarditis (66%; n=59) (Figure 3C). Tuberculous pericarditis is prevalent in South Africa and was responsible for 69.5% (162 of 233) of pericardial effusions in a study done at our institution [20,21]. The most ominous sequela of tuberculous pericarditis is constrictive pericarditis. CMR is valuable in assessing pericardial inflammation and degree of constriction, guiding the decision regarding surgical management (pericardial stripping) in addition to medical management [22].

The diagnostic impact of CMR was high in our study, mirroring the findings of the EuroCMR registry and the GCMR. Cardiovascular magnetic resonance imaging advances patient care by not only improving diagnostic accuracy but also adding valuable information that guides risk stratification and management decisions [7,23].

Although the focus of CMR is the heart, thoracic and abdominal structures near it are also included in the imaging field of view. The frequency of reporting extracardiac incidental findings varies among institutions; however, certain findings are clinically significant, for example, lymphadenopathy reported in our audit [24]. The frequency of incidental extracardiac findings in our audit is lower in comparison to a recent systematic review, suggesting that there is room for improvement in the reporting of extracardiac incidental findings [25].

To the best of our knowledge, this is the first published audit of CMR in sub-Saharan Africa. The CMR research void in sub-Saharan Africa creates a multitude of future research opportunities. At our institution, the CMR service has directly contributed to research output. To date, six doctoral and six master´s research projects have either been completed or are underway (four completed and two in process in each subset); two CMR-related research projects are also ongoing, and 26 CMR-related publications have seen the light.

Internationally there is a drive for CMR audits and registries, but different methodologies and a lack of consistency hamper their generalisability. Retrospective research is often hampered by information bias, especially in CMR in which clinical reports must be read and analyzed with no consistency among these reports. Data collected retrospectively inevitably may lack comprehensiveness and accuracy if the data were collected prior to the inception of the data variable list [8]. This review aims to add to this body of knowledge; however, it represents only a small, selective population from a single institution, and this may decrease its generalisability. This audit does, however, pave the way for a registry of the CMR services provided in the region.

## Conclusion

Establishing a functional CMR service in our resource-poor setting required multidisciplinary collaboration and innovative ideas regarding MRI scanning efficiency. This audit concluded that our CMR service was clinically useful by having a high diagnostic impact in terms of making a new unsuspected diagnosis, confirming a suspected diagnosis, or adding valuable findings influencing patient management. Tygerberg Hospital´s CMR service benefits a unique patient population with a comparatively different distribution of cardiac diseases, which offers diversity to current CMR research. To the best of our knowledge, this is the first CMR service audit in sub-Saharan Africa.

### What is known about this topic

- Cardiovascular magnetic resonance imaging (CMR) is now considered the gold standard in many clinical applications, allowing improved diagnostic accuracy, prognostication, and patient outcome;
- Magnetic resonance imaging (MRI) and especially CMR is a scarce resource in sub-Saharan Africa;
- Performing local and international audits and establishing registries allow optimisation of service quality and standards while aiding in the establishment of evidence-based practice.

### What this study adds

- Establishing a functional CMR service in our resource-poor setting was challenging but possible with multidisciplinary collaboration and innovative ideas;
- Tygerberg Hospital´s CMR service audit produced comparatively different population characteristics and cardiac disease distribution, thus diversifying current research and creating new research opportunities;
- To the best of our knowledge, this is the first published audit of a CMR service in sub-Saharan Africa.
